# Gut microbiota and diet matrix modulate the effects of the flavonoid quercetin on atherosclerosis

**DOI:** 10.21203/rs.3.rs-2431147/v1

**Published:** 2023-01-10

**Authors:** Kazuyuki Kasahara, Robert L. Kerby, Tzu-Wen L. Cross, Jessica Everhart, Colin Kay, Bradley W. Bolling, Fredrik Bäckhed, Federico E. Rey

**Affiliations:** 1 Department of Bacteriology, University of Wisconsin-Madison, Madison, WI, USA; 2 Lee Kong Chian School of Medicine, Nanyang Technological University, Singapore, Singapore; 3 Plants for Human Health Institute, North Carolina State University, Kannapolis, NC, USA; 4 Department of Food Science, University of Wisconsin-Madison, Madison, WI, USA; 5 Wallenberg Laboratory, Department of Molecular and Clinical Medicine, University of Gothenburg, Gothenburg, Sweden

## Abstract

Gut bacterial metabolism of dietary flavonoids results in the production of a variety of phenolic acids, whose contributions to health remain poorly understood. Here, we show that supplementation with the commonly consumed flavonoid quercetin impacted gut microbiome composition and resulted in a significant reduction in atherosclerosis burden in conventionally-raised (ConvR) *Apolipoprotein E* (*ApoE*) knockout (KO) mice fed a high-MAC (microbiota-accessible carbohydrates) diet. However, this effect was not observed in animals consuming a defined diet containing low levels of MAC. Furthermore, we found that the effect of quercetin on atherosclerosis required gut microbes, as supplementation of this flavonoid to germ-free (GF) *ApoE* KO mice consuming the high-MAC diet did not affect the development of atherosclerosis. Metabolomic analysis revealed that consumption of quercetin significantly increased plasma levels of benzoylglutamic acid and protocatechuic acid in ConvR mice exposed to the high-MAC diet, while these increases were not observed in GF mice or conventional animals consuming the low-MAC diet supplemented with the flavonoid. Furthermore, levels of these metabolites were negatively associated with atherosclerosis burden. Altogether, these results suggest that the beneficial effects of quercetin on atherosclerosis are influenced by gut microbes and dietary MAC.

## Introduction

The digestive tract of mammals harbors microbial communities that fulfill important functions including the breakdown of non-digestible carbohydrates (e.g., dietary fiber), shaping of the immune system, and provision of colonization resistance against pathogens^1^. The gut microbiota has also been implicated in regulating host energy metabolism and the onset of various disorders such as inflammatory bowel disease^2^, cancer^3^, and neurological diseases^4^. Recent studies have highlighted the significant roles of the gut microbiota and chronic inflammation in the development of cardiovascular diseases (CVD) including atherosclerosis^5,6,7,8^. A number of bacterial metabolites arising from specific dietary components, including trimethylamine, phenylacetylglutamine , and short-chain fatty acids, have gained recognition as important mediators of cardiovascular health^9–12^.

Epidemiologic studies have linked a high intake of flavonoids—polyphenolic compounds naturally occurring in fruits, vegetables and cereals—with a lower risk of metabolic and cardiovascular diseases. Like dietary proteins and complex plant polysaccharides, flavonoids are also subject to bacterial transformations. Several human studies have linked increased intake of flavonoids with the prevention of cardiovascular diseases^13,14^. This association is supported by animal studies in which the administration of flavonoids as supplements reduces progression of atherosclerosis^15^. Many flavonoids, including the commonly occurring flavonoid quercetin, have been shown to possess anti-oxidative and anti-inflammatory activity^16^. In general, dietary quercetin and other flavonoids are not efficiently absorbed in the proximal small intestine, with a significant fraction of these compounds reaching the distal small intestine and the colon, where they are metabolized by the gut microbiota^17,18^. Gut bacterial metabolism of quercetin and other flavonoids results in several phenolic acids that may exert beneficial effects on the host. Exposure to quercetin through diet is also known to impact gut microbiome composition^19,20^.

Despite the evidence suggesting that quercetin supplementation has protective effects on atherosclerosis and modifies gut microbiota composition^21^, it is still unknown whether the gut microbiota contributes to the beneficial effects of this flavonoid on vascular disease. In addition, the bioavailability of flavonoids varies depending on the food matrix^22^. Quercetin is lipophilic, and diets with high lipid content enhance its absorption^23^. However, it is not known whether other food components, such as plant polysaccharides, which largely co-occur with flavonoids and modulate the gut microbiome, affect the beneficial effects of this flavonoid on atherosclerosis. Interestingly, a meta-analysis of human trials showed considerable inter-individual variability of cardiometabolic biomarkers in response to flavonoid supplementation^24^, which may be associated with inter-individual differences in the gut microbiome and diet. In this study, we assessed the influence of the gut microbiota and diet matrix on quercetin-mediated athero-protection. Our results suggest that bacterial metabolism and complex plant polysaccharides modulate the protective effect of quercetin on atherosclerosis.

## Results

### Diet matrix modulates the effects of quercetin on atherosclerosis.

**Figure 1.**
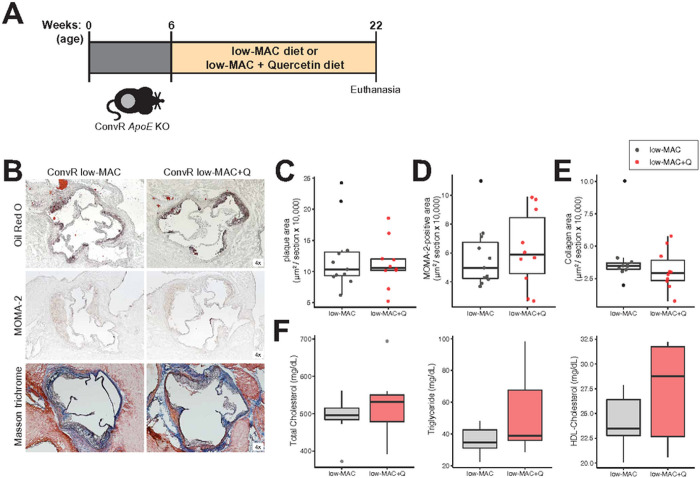
Dietary quercetin does not affect atherosclerosis development in mice fed a low-MAC diet. **A)** Experimental design. **B-E)** Representative sections and quantitative analyses of Oil Red O staining (B,C; n=11 in the ConvR/low-MAC group and n=10 in the ConvR/low-MAC+Q group), MOMA-2 staining (B,D; n=11 in the ConvR/low-MAC group and n=10 in the ConvR/low-MAC+Q group), and Masson’s trichrome staining (B,E; n=11 in the ConvR/low-MAC group and n=10 in the ConvR/low-MAC+Q group) in the aortic sinus. The data were expressed as box-and-whisker plots, where the boxes indicate the median values and the interquartile ranges, and the whiskers represent the minimum and maximum values. **F)** Plasma lipid profiles (n=7 in the ConvR/low-MAC group and n=7 in the ConvR/low-MAC+Q group). Unpaired two-tailed Student’s t-test were performed. MAC; microbiota-accessible carbohydrates, *ApoE*; *Apolipoprotein E*, ConvR; conventionally-raised, Q; quercetin, MOMA; monocytes and macrophages.

**Figure 2.**
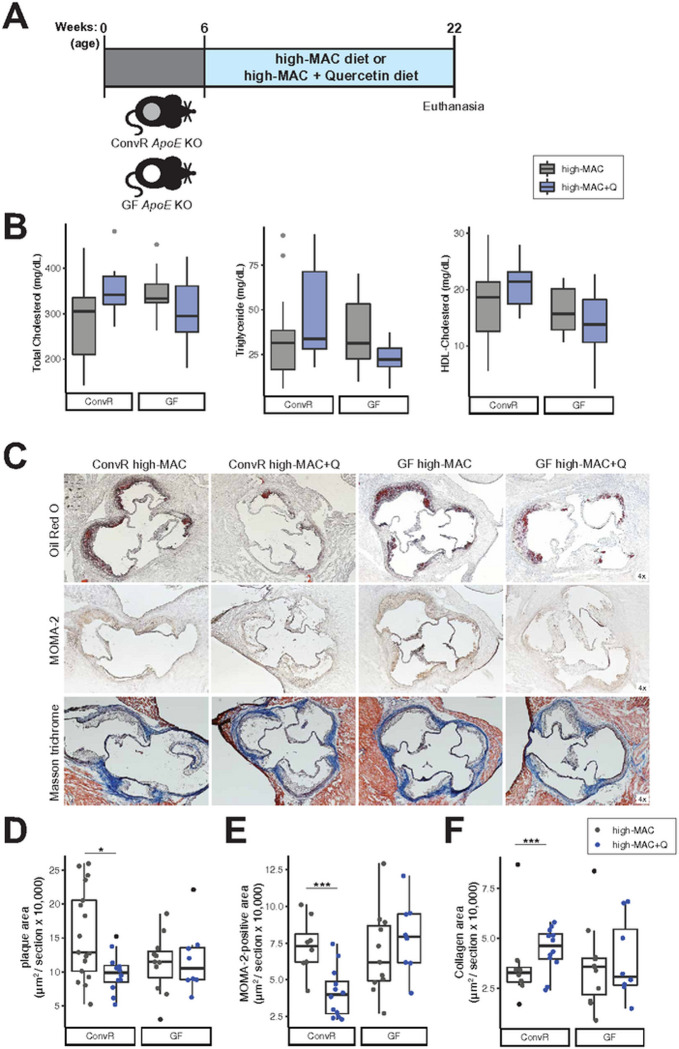
The gut microbiota mediates the athero-protective effect of quercetin in mice fed a high-MAC diet. **A)** Experimental design. **B)** Plasma lipid profiles (n=17 in the ConvR/high-MAC group, n=12 in the ConvR/high-MAC+Q group, n=11 in the GF/high-MAC group, and n=8 in the GF/high-MAC+Q group). **C-F)** Representative sections and quantitative analyses of Oil Red O staining (C,D; n=17 in the ConvR/high-MAC group, n=12 in the ConvR/high-MAC+Q group, n=11 in the GF/high-MAC group, and n=8 in the GF/high-MAC+Q group), MOMA-2 staining (C,E; n=8 in the ConvR/high-MAC group, n=12 in the ConvR/high-MAC+Q group, n=11 in the GF/high-MAC group, and n=8 in the GF/high-MAC+Q group), and Masson’s trichrome staining (C,F; n=8 in the ConvR/high-MAC group, n=12 in the ConvR/high-MAC+Q group, n=11 in the GF/high-MAC group, and n=8 in the GF/high-MAC+Q group) in the aortic sinus. The data were expressed as box-and-whisker plots, where the boxes indicate the median values and the interquartile ranges and the whiskers represent the minimum and maximum values. Significance was calculated by two-way ANOVA with Bonferroni post-tests as follows: *, *P* value of <0.05; ***, *P* value of <0.001. MAC; microbiota-accessible carbohydrates, *ApoE*; *Apolipoprotein E*, ConvR; conventionally-raised, GF; germ-free, Q; quercetin, MONA; monocytes and macrophages.

We tested the effect of quercetin on atherosclerosis progression in mice fed a low-fat low-MAC (microbiota-accessible carbohydrates) diet. Conventionally-raised (ConvR) *Apolipoprotein E* (*ApoE*) knockout (KO) mice were fed a low-MAC diet or a low-MAC diet supplemented with 0.1% w/w quercetin (Suppl. Table 1) starting at 6-week-old and maintained in the diet for 16 weeks. Atherosclerosis burden was analyzed in tissue collected from 22-week-old animals (Fig. 1A). Unexpectedly, quercetin did not affect plasma lipid profile, atherosclerosis lesion size, and macrophage or collagen levels in the aortic sinus (Fig. 1B–1F). Since it has been reported that dietary fiber affects the bioavailability of phenolic compounds^22^, we assessed whether quercetin exerted protective effects against atherosclerosis when supplemented into a grain-based high-MAC diet containing a relatively low-fat content (18% calories derived from fat, Suppl. Table 2). Six-week-old ConvR *ApoE* KO mice were fed the high-MAC diet supplemented with 0.1% quercetin or the same diet without the flavonoid (control) for 16 weeks (Fig. 2A). Once again quercetin supplementation did not affect plasma lipid profiles in these mice (Fig. 2B). Nonetheless, quercetin significantly reduced atherosclerotic lesion size (aortic sinus median plaque area: 12.9×10^4^ μm^2^ [control diet] *vs.* 9.8×10^4^ μm^2^ [quercetin-supplemented diet], Fig. 2C, D). Immunohistochemical studies of atherosclerotic lesions showed that mice fed the high-MAC diet supplemented with quercetin developed atherosclerotic lesions that contained a lower number of macrophages (Fig. 2C, E) and increased levels of collagen (Fig. 2C, F), suggesting that quercetin supplementation reduced aortic inflammation and promoted the stability of atherosclerotic plaques in the presence of dietary plant polysaccharides.

### Quercetin modulates gut microbiome composition and the gut microbiome mediates the beneficial effects of quercetin on atherosclerosis.

**Fig. 3:**
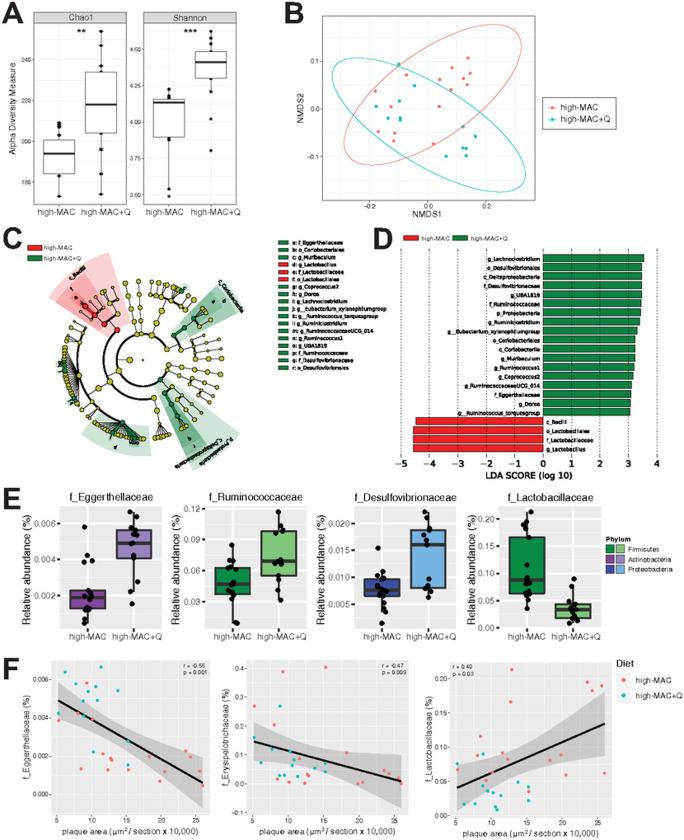
Supplementation of quercetin modulates gut microbiota composition in ConvR mice fed a high-MAC diet. **A)** Alpha diversity of gut microbial communities assessed by Chao1 and the Shannon index (t-test; **, *P* value of <0.01; ***, *P* value of <0.001). **B)** Non-metric multidimensional scaling (NMDS) plot of weighted UniFrac analysis of relative sample ASV composition with the PERMANOVA test showing a significant influence of quercetin on microbial community composition. **C)** Cladogram generated from LEfSe analysis showing the relationship between taxa (the levels represent, from the inner to outer rings, phylum, class, order, family, and genus). **D)** Linear discriminant analysis (LDA) scores derived from LEfSe analysis, showing the biomarker taxa (LDA score [log 10] of >3 and a significance of *P* < 0.05 determined by the Wilcoxon signed-rank test). **E)** Bacterial families differentially represented in cecal contents from the high-MAC+Q mice compared to the control group (*P* value of <0.05, FDR-corrected). **F)** Correlation of bacterial families with atherosclerotic plaque area. Pearson’s rho and *P* values were calculated by Pearson correlation coefficient. n=17 in the ConvR/high-MAC group and n=12 in the ConvR/high-MAC+Q group. MAC; microbiota-accessible carbohydrates, ConvR; conventionally-raised, Q; quercetin, ASV; amplicon sequence variant.

To investigate whether the effect of quercetin on atherosclerosis is associated with changes in gut microbiota composition, we characterized the cecal microbiomes of the *ApoE* KO mice discussed above using 16S rRNA gene sequencing. It is important to note that for each treatment condition, mice were distributed in multiple cages (3–6 cages/group). We found that mice consuming high-MAC plus quercetin showed significantly increased richness of the gut microbiota as determined by the Chao1 index (Fig. 3A), which uses a nonparametric model to calculate a conservative estimate of total amplicon sequence variant (ASV) richness for each sample. In contrast, quercetin did not change the Chao1 index in mice consuming low-MAC diet (**Suppl. Fig. 2A**). However, quercetin-fed animals harbored more diverse microbiomes as determined by the Shannon index both in the high-MAC- and low-MAC-fed animals (Fig. 3A, **Suppl. Fig. 2A**). Non-metric multidimensional scaling (NMDS) analysis of weighted UniFrac^25^ distances revealed a significant influence of quercetin (PERMANOVA; *P* = 0.017) on microbial community composition in mice consuming the high-MAC diet (Fig. 3B). Also, linear discriminant analysis (LDA) effect size (LEfSe Galaxy Version 1.0)^26^ was performed to identify taxonomic differences in microbiota composition between the two groups of mice. Fig. 3C illustrates the differential phylogenetic distributions of microbial communities in these two groups. Taxa belonging to the Eggerthellaceae, Ruminococcaceae, and Desulfovibrionaceae families and the genus *Parvibacter*, *Dorea*, and *Ruminiclostridium* were increased in the quercetin-fed mice relative to control mice (Fig. 3C, 3D, **Suppl. Fig. 1A**), whereas the members of the Lactobacillaceae family were present at lower levels in the presence of the flavonoid (LDA score [log 10] > 4, Fig. 3D). In mice consuming the low-MAC diet, quercetin also impacted microbial community composition (**Suppl. Fig. 2B**) and phylogenetic distributions of microbial communities (**Suppl. Fig. 2C, 2D**), but distinct microbial taxa between the two diets were changed by quercetin supplementation (**Suppl. Fig. 2E**). Interestingly, quercetin increased the genus *Eubacterium xylanophilum* group and the Eggerthellaceae family both in mice consuming the low-MAC and the high-MAC diets, however its abundance was lower in mice fed the low-MAC diet. In mice fed the high-MAC diet, atherosclerotic plaque areas were negatively associated with the Eggerthellaceae and Erysipelotrichaceae families and positively associated with the Lactobacillaceae family (Fig. 3F). Collectively, dietary quercetin increased bacterial richness and modified several microbial taxa associated with atherosclerosis in mice fed the high-MAC diet.

Given the observed changes in gut microbiota composition in response to quercetin, we next examined whether the gut microbiota modulated the protective effects of this flavonoid on atherosclerosis. Germ-free (GF) *ApoE* KO mice were fed the high-MAC diet with or without quercetin for 16 weeks (Fig. 2A). In contrast to the observations made in ConvR mice, we found that quercetin supplementation did not affect atherosclerotic lesion size, aortic macrophage area, or collagen levels in GF mice fed the high-MAC diet (Fig. 2C–2F). Lipid profiles were also not impacted by quercetin in GF mice. Altogether, these results suggest that the athero-protective effects of quercetin depend on the presence of gut microbiota.

### Microbial phenolic metabolites in blood are associated with atheroprotection.

Bacterial fermentation of MACs results in the production of short-chain fatty acids (SCFAs), including acetate, propionate, and butyrate, which have associated with athero-protection^9,12^. Previous work suggests that flavonoids may influence production of SCFAs^27^. To start exploring potential mechanisms by which quercetin inhibits the development of atherosclerosis we measured levels of SCFAs in cecal contents. Quercetin did not change cecal levels of acetate, propionate, and butyrate in ConvR mice consuming either the high- or low-MAC diet (**Suppl. Fig. 3**).

**Figure 4.**
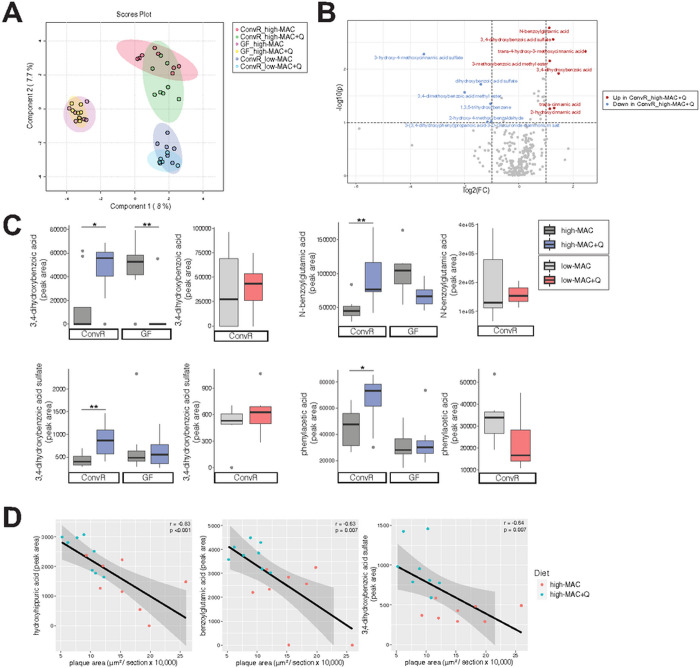
Plasma metabolites derived from quercetin are associated with athero-protective effects. **A)** Partial Least Squares Discriminant Analysis (PLS-DA) plot based on the data derived from the targeted metabolomics of plasma in the ConvR mice and the GF mice. (n=8 in the ConvR/HPP high-MAC group, n=8 in the ConvR/high-MAC+Q group, n=7 in the GF/high-MAC group, n=7 in the GF/high-MAC+Q group, n=6 in the ConvR/low-MAC group, n=6 in the ConvR/low-MAC+Q group). **B)** Volcano plot of metabolites in the ConvR/high-MAC vs ConvR/high-MAC+Q group, with log-transformed adjusted *P* values and fold changes. Red circles; increased in the ConvR/high-MAC+Q group. Blue circles; increased in the ConvR/high-MAC group. **C)** The values for 3,4-dihydroxybenzoic acid and its sulfate, N-benzoylglutamic acid, and phenylacetic acid were expressed as box-and-whisker plots. Significance was calculated by two-way ANOVA (high-MAC) and unpaired two-tailed Student’s t-test (low-MAC) with the Benjamini-Hochberg correction as follows; *, *P* value of <0.05; **, *P* value of <0.01. **D)** Correlation of phenols with atherosclerotic plaque area. Pearson’s rho and *P* values were calculated by Pearson correlation coefficient. MAC; microbiota-accessible carbohydrates, ConvR; conventionally-raised, GF; germ-free, Q; quercetin.

We next analyzed phenolic metabolites in plasma samples using Ultra Performance Liquid Chromatography-Tandem Mass Spectrometer (UPLC-MS/MS). Partial Least Squares Discriminant Analysis (PLS-DA) plot showed significant separation among low-MAC-fed ConvR, high-MAC-fed ConvR, and high-MAC-fed GF mice, with modest separation between control and quercetin-supplemented diet in the high-MAC and the low-MAC ConvR mice. Interestingly, there was no separation between GF controls and GF quercetin animals (Fig. 4A). We also determined levels of quercetin and its derivatives (quercetin 3-O-glucuronide, quercetin 3-O-sulfate, isorhamnetin glucuronide) in the circulation. Unexpectedly, there was little to no changes in those metabolites (**Suppl. Fig. 4**), suggesting that quercetin was further metabolized by gut microbes. Comparison of phenolic metabolites in ConvR mice consuming high-MAC *vs*. high-MAC+Q showed that several metabolites, such as benzoylglutamic acid, 3,4-dihydroxybenzoic acid (protocatechuic acid) and its sulfate form, trans-4-hydroxy-3-methoxycinnamic acid (ferulic acid), and 3-methoxybenzoic acid methyl ester, were significantly increased by the quercetin supplementation (Fig. 4B). This was also confirmed by Variable Importance in Projection (VIP) scores (**Suppl. Fig. 5A**) and correlation coefficients (**Suppl. Fig. 5B**). Interestingly, quercetin supplementation did not increase these metabolites in GF mice or ConvR mice with the low-MAC diet (Fig. 4C), suggesting that quercetin requires both the gut microbiota and dietary MAC to increase these phenolic metabolites. Moreover, atherosclerotic plaque areas from the ConvR mice consuming high-MAC diets (plus/minus quercetin) were negatively associated with hydroxyhippuric acid, benzoylglutamic acid, and 3,4-hydroxybenzoic acid sulfate (protocatechuic acid-sulfate) (Fig. 4D). Collectively, these results suggested that dietary quercetin increased several plasma phenolic metabolites derived of bacterial metabolism including protocatechuic acid, when provided in concert with dietary plant polysaccharides.

## Discussion

A large body of literature supports the notion that consumption of dietary flavonoids decreases the risk of cardiovascular diseases^14^, and that consumption of flavonoids is associated with changes in the gut microbiome^28^. The food matrix is also an important factor affecting the bioaccessibility and bioavailability of flavonoids^22^. Our study provides causal evidence linking the effect of quercetin consumption on atherosclerosis with the gut microbiome and food matrix.

Flavonoids are metabolized by phase I and phase II metabolism in the intestine and liver. In the colon, resident gut bacteria can convert unabsorbed flavonoids into small phenolic acids and aromatic metabolites^29,30^. The effects these metabolites have on the host are poorly described. Feeding studies with tracing of metabolic conversion suggest flavonoid catabolites are readily absorbed in the colon, often possess longer half-lives and reach substantially higher systemic concentrations than parent compounds^31^. These observations have increased the interest in microbiota-generated metabolites, which might mediate cardiometabolic effects of flavonoids. Degradation of quercetin by the gut microbiota involves C-ring fission, formation of 3-(4-hydroxyphenyl)propionic acid, and subsequent transformation to 3,4-dihydroxyphenylacetic acid^32^. Further modification leads to 3,4-dihydroxybenzoic acid (protocatechuic acid) and 4-hydroxybenzoic acid. 3,4-dihydroxyphenylacetic acid can also be dehydroxylated to 3-hydroxyphenylacetic acid or 4-hydroxyphenylacetic acid and phenylacetic acid, further degrading into various smaller products^33^. Our semi-quantitative targeted phenol metabolomic analysis identified several microbiota-generated metabolites from quercetin, such as protocatechuic acid, and ferulic acid that were elevated in plasma from animals consuming high-MAC supplemented with quercetin. These results are consistent with previous findings showing that protocatechuic acid and ferulic acid are protective against atherosclerosis development in animal models^34,35^, whereas the effect of benzoylglutamic acid (also increased by quercetin in mice fed high MAC diet) on atherogenesis has not been explored. Importantly, these metabolites were not increased by quercetin consumption in GF mice or mice consuming the low-MAC diet, emphasizing the role of the gut microbiota and dietary plant polysaccharides in the generation of these metabolites. Thus, we identified several microbiota-generated metabolites from quercetin that were associated with the protection against atherosclerosis.

Dietary quercetin can alter gut microbial composition partly because of probiotic-like properties and stimulation of growth of specific bacteria^27^. Similarly, our 16S rRNA sequencing data showed that quercetin increased microbiota richness and alpha diversity. Eggerthellaceae, Ruminococcaceae, and Desulfovibrionaceae families were highly enriched in the quercetin-fed high-MAC mice. However, whether these taxa have the capacity to degrade quercetin is still unknown. Interestingly, *Ellagibacter isourolithinifaciens* belonging to the Eggerthellaceae family, a recently isolated bacterium from human feces, can metabolize ellagic acid into isourolithin A so that the taxa in the Eggerthellaceae family would potentially metabolize quercetin in the gut^36^. Using a GF mouse model of atherosclerosis, we found that the gut microbiota is responsible for the protective effect of quercetin against the disease. Future studies using gnotobiotic mice colonized with a defined consortium of microbes will help clarify the role of flavonoid-metabolizing bacteria on host physiology and disease.

Flavonoids are commonly mixed with different macromolecules including carbohydrates, lipids, and proteins that affect their bioaccessibility (i.e., amount of an ingested nutrient available for absorption in the gut after digestion) and bioavailability (i.e., proportion that is digested, absorbed, and used)^22^. While the protective effects of quercetin on atherosclerosis have been previously described in mice^15,21,37,38^, in most cases, western-type diets (i.e., high-fat, high-cholesterol diets) were used to exacerbate disease. Quercetin is lipophilic, and the high lipid content in these diets enhances the efficiency of quercetin absorption^23^. This may explain why we did not observe a reduction in atherosclerosis in mice fed the low-fat, low-MAC diet. Furthermore, our results suggest that quercetin’s effect on atherosclerosis is influenced by dietary plant polysaccharides. Although we did not provide the mechanisms by which the dietary plant polysaccharides impact quercetin to exert its action, it has been shown that they prolong gastric emptying time and delay absorption of flavonoids. In addition, dietary fiber may reduce rates of flavonoid absorption mainly by physically trapping the flavonoids within the fiber matrix in the chyme^22^.

In summary, we show that the protective effect of quercetin on atherosclerosis depends on the gut microbiota and dietary matrix, potentially complex plant polysaccharides which are associated with increased accumulation of phenolic acids in the blood. Further studies are warranted to clarify the metabolic processes underlying the generation of specific bioavailable, bioactive phenolic acid metabolites and to identify bacteria consortiums that optimize the generation of these phenolic acids. These studies will facilitate the development of symbiotic approaches for preventing cardiovascular diseases.

## Methods

### Gnotobiotic husbandry.

All GF C57BL/6 and *ApoE* KO mice were maintained in a controlled environment in plastic flexible film gnotobiotic isolators under a strict 12h light/dark cycle and received sterilized water and standard chow (LabDiet 5021; LabDiet, St Louis, MO) *ad libitum* until 6 weeks of age. Using traditional microbiology methods, the sterility of GF animals was assessed by incubating freshly collected fecal samples under aerobic and anaerobic conditions.

### Animals and experimental design.

Experiments: i) Six-week-old male ConvR *ApoE* KO mice were fed a defined diet composed of 17.7% (w/w) protein, 60.1% carbohydrate, and 7.2% fat (i.e., low-MAC diet, TD.97184; Envigo, Suppl. Table 1) or the low-MAC diet supplemented with 0.1% (w/w) quercetin (TD.150881; Envigo, Suppl. Table 2) for 16 weeks. ii) Six-week-old male ConvR or GF C57BL/6 *ApoE* KO mice were fed a standard grain-based chow diet (i.e., high-MAC diet, TD.2018; Envigo, Suppl. Table 2) or the high-MAC diet supplemented with 0.1% (w/w) quercetin (TD.150883; Envigo, Suppl Table 2) for 16 weeks. Dietary fiber in the high-MAC diet is derived from various plants, including ground wheat, ground corn, wheat middling, dehulled soybean meal, and corn gluten meal. Mice were then euthanized at 22 weeks of age after 4h fasting. The experimental diets were sterilized by irradiation. Mice were then euthanized at 22 weeks of age after 4h fasting. All experiments were performed using protocols approved by the University of Wisconsin-Madison Animal Care and Use Committee.

### Atherosclerotic lesion assessments.

Atherosclerotic lesions were assessed as previously described. Briefly, mice were anaesthetized, and the aorta was perfused with PBS. To determine the atherosclerotic lesion size at the aortic sinus, the samples were cut in the ascending aorta, and the proximal samples containing the aortic sinus were embedded in OCT compound (Tissue-Tek; Sakura Finetek, Tokyo, Japan). Five consecutive sections (10 μm thickness) taken at 100 μm intervals (i.e. 50, 150, 250, 350, and 450 μm from the bottom of the aortic sinus) were collected from each mouse and stained with Oil Red O. The atherosclerosis volume in the aortic sinus was expressed as the mean size of the 5 sections for each mouse. Immunohistochemistry was performed on formalin-fixed cryosections of mouse aortic roots using antibodies to identify macrophages (MOMA-2, 1:50; ab33451, Abcam, Cambridge, MA), followed by detection with biotinylated secondary antibodies (1:400; ab6733, Abcam) and streptavidin-horseradish peroxidase (1:500; P0397, Dako, Carpinteria, CA). Negative controls were prepared with substitution with an isotype control antibody. Staining with Masson’s trichrome was used to delineate the fibrous area according to the manufacturer’s instructions (ab150686, Abcam). Stained sections were digitally captured, and the stained area was calculated. Plaque area, Oil Red O-positive area, macrophage area, and fibrous area were measured using Image J software (National Institutes of Health, Bethesda, MD).

### DNA extraction from cecal contents.

DNA was isolated from feces by extraction using a bead-beating protocol^9^. Mouse cecal samples were re-suspended in a solution containing 500μl of extraction buffer [200mM Tris (pH 8.0), 200mM NaCl, 20mM EDTA], 210μl of 20% SDS, 500μl phenol:chloroform:isoamyl alcohol (pH 7.9, 25:24:1) and 500μl of 0.1-mm diameter zirconia/silica beads. Cells were mechanically disrupted using a bead beater (BioSpec Products, Barlesville, OK; maximum setting for 3 min at room temperature), centrifuged to separate phases, then the nucleic acids in the aqueous phase was precipitate by addition of isopropanol. Following solubilization in 10 mM Tris/HCl (pH 8.0) + 1 mM EDTA, contaminants were removed using QIAquick 96-well PCR Purification Kit (Qiagen, Germantown, MD, USA). Isolated DNA was eluted in 5 mM Tris/HCL (pH 8.5) and was stored at −80°C until further use.

### 16S rRNA gene sequencing.

PCR was performed using universal primers flanking the variable 4 (V4) region of the bacterial 16S rRNA gene^39^. Genomic DNA samples were amplified in duplicate. Each reaction contained 25 ng genomic DNA, 10 μM of each uniquely barcoded primer, 12.5 μl 2x HiFi HotStart ReadyMix (KAPA Biosystems, Wilmington, MA, USA), and water to a final reaction volume of 25 μl. PCR was carried out under the following conditions: initial denaturation for 3 min at 95°C, followed by 20 cycles of denaturation for 30 s at 95°C, annealing for 30 s at 55°C and elongation for 30 s at 72°C, and a final elongation step for 5 min at 72°C. PCR products were purified with the QIAquick 96-well PCR Purification Kit and quantified using the Qubit dsDNA HS Assay kit (Invitrogen, Oregon, USA). Samples were equimolar pooled and sequenced by the University of Wisconsin–Madison Biotechnology Center with the MiSeq 2×250 v2 kit (Illumina, San Diego, CA, USA) using custom sequencing primers.

### Microbiota analysis in QIIME2.

Demultiplexed paired-end fastq files were generated by CASAVA (Illumina), and a sample mapping file were used as input files. Sequences were processed, quality filtered and analyzed with QIIME2 (version 2019.10) (https://qiime2.org), a plugin-based microbiome analysis platform^40^. DADA2^41^ was used to denoise sequencing reads with the q2-dada2 plugin for quality filtering and identification of amplicon sequence variants (ASVs) (i.e. 100% exact sequence match). This resulted in 3,580,038 total sequences, with an average of 81,364 sequences per sample. Sequence variants were aligned with mafft^42^ with the q2-alignment plugin. The q2-phylogeny plugin was used for phylogenetic reconstruction via FastTree^43^. Taxonomic classification was assigned using classify-sklearn^44^ against the SILVA 132 reference sequences^45^. Alpha- and beta-diversity (weighted and unweighted UniFrac^25^) analyses were performed using the q2-diversity plugin at a rarefaction depth of 30000 sequences per sample. Subsequent processing and analysis were performed in R (v.3.6.2), and data generated in QIIME2 was imported into R using Phyloseq^46^. LefSe analysis was performed using parameters as follows (p < 0.05 and LDA score 3.0;^26^).

### Plasma biochemical analysis.

Blood samples were drawn by cardiac puncture under anesthesia using isoflurane. Plasma was acquired by centrifugation and stored at −80°C until measurement. The triglycerides, total cholesterol, and high-density lipoprotein cholesterol levels were measured with commercially available kits from Wako Chemicals (Richmond, VA).

### GC-MS of short-chain fatty acid measurement.

Sample preparation was based on a previously described procedure^47^, with some modifications. Cecal contents were weighed in 4mL vials, then 10 μL of a mixture of internal standards (20 mM each; acetic acid-D4, Sigma-Aldrich #233315; propionic acid-D6, Sigma-Aldrich #490644; and butyric acid-D7, CDN isotopes #D-171) was subsequently added, followed by 20 μL of 33% HCl and 1 mL diethyl ether and the vials were sealed with polytetrafluoroethylene-lined screw caps. For plasma samples, 50 μL of each sample, 1.25 μL of the internal standard mix, 5 μL of 33% HCl, and 0.75 mL of diethyl ether were mixed. The mixture was vortexed vigorously for 3 min and then centrifuged (4,000 ×*g*, 10 min). The upper organic layer was transferred to another vial, and a second diethyl ether extraction was performed. After combining the two ether extracts, a 60 μL aliquot was removed, combined with 2 μL *N-tert-*butyldimethylsilyl-*N*-methyltrifluoroacetamide (MTBSTFA, Sigma-Aldrich #394882) in a GC auto-sampler vial with a 200 μL glass insert, and incubated for 2 h at room temperature. Derivatized samples (1 μL) were injected onto an Agilent 7890B/5977A GC/MSD instrument with Agilent DB1-ms 0.25 mm × 60 m column with a 0.25 μm bonded phase. A discontinuous oven program was used, starting at 40°C for 2.25 min, then ramping at 20°C/min to 200°C, then ramping at 100°C/min to 300°C and holding for 7 min. The total run time was 18.25 minutes. Linear column flow was maintained at 1.26mL/min. The inlet temperature was set to 250°C with an injection split ratio of 15:1. Quantitation was performed using selected ion monitoring (SIM) acquisition mode, and metabolites were compared to relevant labelled internal standards using Agilent Mass Hunter v. Acquisition B.07.02.1938. The m/z of monitored ions are as follows: 117 (acetic acid), 120 (acetic acid-D4), 131 (propionic acid), 136 (propionic acid-D6), 145 (butyric acid), and 151 (butyric acid-D7). Concentrations were normalized to mg of cecal contents.

### Targeted phenol metabolome for plasma samples.

The UPLC-MS/MS advanced scheduled multiple-reaction monitoring (ADsMRM) scanning methodological workflow was utilized to identify metabolites of quercetin (quercetin, isorhamnetin, quercetin-sulfate, quercetin-glucuronide), along with other phytochemical and host metabolites which may be impacted by treatment with quercetin. The metabolites were purified from 100 μl plasma by 96-well plate solid phase extraction (SPE; Strata^™^-X Polymeric Reversed Phase, microelution 2 mg/well). The solid phase extraction treated samples were chromatographically separated and quantified using Exion high-performance liquid chromatography-tandem hybrid triple quadrupole-linear ion trap mass spectrometer (SCIEX QTRAP 6500+; UHPLC-ESI-MS/MS) with electrospray IonDrive Turbo-V Source. The samples were injected into a Kinetex PFP UPLC column (1.7 μm particle size, 100 Å pore size, 100 mm length, 2.1 mm internal diameter; Phenomenex^®^) with oven temperature maintained at 37°C. Mobile phase A and mobile phase B consisted of 0.1% v.v. formic acid in water and 0.1% v.v. formic acid in LC-MS grade acetonitrile, with a binary gradient ranging from 2% B to 90% B over 30 min and flow rate gradient from 0.55 mL/min to 0.75 mL/min. MS/MS scanning was accomplished by ADsMRM using polarity switching between positive and negative ionization mode in Analyst (v.1.6.3, SCIEX) and with peak area and intensity recorded using SCIEX OS (v.2.0.0.45330, SCIEX). Internal standards included L-tyrosine-13C9,15N, resveratrol-13C6, hippuric acid 13C, 13C6 4-hydroxybenzoic acid propyl ester, and phlorizin dehydrate (Sigma). Peaks matching retention time, fragmentation patterns, and having intensity greater than 1e4, area greater than 2e4, and number of data points across baseline greater than 5 were annotated, and peak area, height, and area:height ratio were returned for statistical analysis.

### Metabolome Analysis.

Metabolites and their respective normalized peak areas were analyzed by the MetaboAnalystR package. Partial least square-discriminant analysis (PLS-DA) was used to determine the separation between groups of the metabolite variables through rotation of the principal components obtained by PCA. Volcano plots were used to compare the size of the fold change to statistical significance. Volcano plots of significantly changing metabolites were determined using a two-sample Student’s t-test with a probability threshold of *P* <0.05 corrected for multiple comparisons using the false discovery rate for type-1 error control.

### Statistical Analysis.

The data were expressed as individual dots with mean ± SEM or box-and-whisker plots where the center line was the median, boxes extended to 25^th^ and 75^th^ percentiles, and whiskers extended to min and max values, and analyzed using R (3.6.2). For the low-MAC diet, significant differences between two groups were evaluated by two-tailed unpaired Student’s t-tests. For the high-MAC diet, significance was calculated by two-way ANOVA with Bonferroni post-tests. Correlation between two variables was calculated by Pearson correlation coefficient. Linear discriminant analysis effect size (LefSe) used a nonparametric Wilcoxon sum-rank test followed by LDA analysis to measure the effect size of each abundant taxon, and two filters (*P* < 0.05 and LDA score of >3) were applied to the present features.

## Supplementary Material

Supplement 1

Supplement 2

## Data Availability

The 16S rRNA sequencing data are available from the Sequence Read Archive (SRA) under accession PRJNA904065.
